# An Example of Compulsory Vaccination

**Published:** 1902-05-10

**Authors:** 


### AN EXAMPLE OF COMPULSORY VACCINATION.

In October, 1898, small-pox was endemic in Porto Rico ; in December it was epidemic ; in January, 1899, it had spread over the island, and by February there were over 3,000 recent cases. Under these circumstances the Americans having recently taken up "the white man's burden," determined to vaccinate the whole population. In February, the work was begun in all parts of the island,[1] and was vigorously carried for four months, until by July 1st 860,000 vaccinations had been done in a population of about 960,000 persons. Of these 87½ per cent. were successful. The work then ceased ; the disease had practically disappeared. In the two and a-half years that have since passed, instead of the former annual average death rate of 621, the mortality from small-pox has been but two per annum. Here we have a good example of the grasp of principles and the thoroughness of method which are so characteristic of the inhabitants of the United States in all they undertake. The present position of vaccination in England is rapidly becoming as intolerable as it has long been inexcusable. If we spend enormous sums every year upon vaccination surely that is done in order that we may obtain protection from small-pox. Yet that object seems in practice to have been entirely put on one side, the trouble and the money which vaccination costs being so expended as to give in return the least possible protection. Truly we are a long-suffering and stupid people. For how many months have we not had this wretched small-pox wandering about London and the neighbourhood, and how many hundreds of thousands of pounds have we not already spent upon small-pox hospitals—a totally unnecessary and wasteful expenditure which we owe entirely to the anti-vaccinists ? The Americans did not worry about hospitals. They knew just what we know; but they had the energy and the will which we have not, and while we discuss the threadbare topic in the papers, they stamp out the epidemic. They acted according to their knowledge. Here is a sample of their method, an extract from the circular by which the inhabitants of the island were finally converted to the advantages of vaccination. "No person who cannot present a duly attested official certificate of vaccination after the date when the official vaccination in his or her barrio or district is completed, shall be admitted to any school, public or private; shall travel by any public conveyance, visit any theatre or any place of public resort, engage in any occupation related to the public, or receive employment. All school teachers, managers, employers and others affected by this order, will govern themselves accordingly under penalty." Would that the Americans, as well as "electrifying" and "tubing" us, would kindly undertake our vaccination.

[1] New York Medical News, April 19.

